# Cytoskeleton Modifications and Autophagy Induction in TCam-2 Seminoma Cells Exposed to Simulated Microgravity

**DOI:** 10.1155/2014/904396

**Published:** 2014-07-17

**Authors:** Francesca Ferranti, Maria Caruso, Marcella Cammarota, Maria Grazia Masiello, Katia Corano Scheri, Cinzia Fabrizi, Lorenzo Fumagalli, Chiara Schiraldi, Alessandra Cucina, Angela Catizone, Giulia Ricci

**Affiliations:** ^1^Italian Space Agency (ASI), Via del Politecnico snc, 00133 Rome, Italy; ^2^Department of Anatomy, Histology, Forensic Medicine and Orthopedics, Sapienza University of Rome, Viale Regina Elena 336, 00161 Rome, Italy; ^3^Department of Experimental Medicine, Second University of Naples, Via Santa Maria di Costantinopoli 16, 80138 Naples, Italy; ^4^Department of Clinical and Molecular Medicine, Sapienza University of Rome, Viale Regina Elena 291, 00161 Rome, Italy; ^5^Systems Biology Group, Sapienza University of Rome, Via A. Scarpa 16, 00161 Rome, Italy; ^6^Department of Surgery “Pietro Valdoni,” Sapienza University of Rome, Viale del Policlinico 155, 00161 Rome, Italy

## Abstract

The study of how mechanical forces may influence cell behavior via cytoskeleton remodeling is a relevant challenge of nowadays that may allow us to define the relationship between mechanics and biochemistry and to address the larger problem of biological complexity. An increasing amount of literature data reported that microgravity condition alters cell architecture as a consequence of cytoskeleton structure modifications. Herein, we are reporting the morphological, cytoskeletal, and behavioral modifications due to the exposition of a seminoma cell line (TCam-2) to simulated microgravity. Even if no differences in cell proliferation and apoptosis were observed after 24 hours of exposure to simulated microgravity, scanning electron microscopy (SEM) analysis revealed that the change of gravity vector significantly affects TCam-2 cell surface morphological appearance. Consistent with this observation, we found that microtubule orientation is altered by microgravity. Moreover, the confocal analysis of actin microfilaments revealed an increase in the cell width induced by the low gravitational force. Microtubules and microfilaments have been related to autophagy modulation and, interestingly, we found a significant autophagic induction in TCam-2 cells exposed to simulated microgravity. This observation is of relevant interest because it shows, for the first time, TCam-2 cell autophagy as a biological response induced by a mechanical stimulus instead of a biochemical one.

## 1. Introduction

An increasing number of experimental observations have demonstrated that tissue homeostasis could be strongly influenced and regulated by physical forces, such as the modulation of gravity vector. In the recent years, many efforts have been made to elucidate the effect of microgravity on cell behavior, and accumulating data show that microgravity alters, permanently or transiently, important biological processes such as mitosis, differentiation, survival, cell morphology, and gene expression profiles [1–7]. However, how cells sense these signals and convert them into a biochemical response remains an important question that needs to be addressed. Recent studies have focused on the cytoskeleton as initial gravity sensor [1, 8]. Cytoskeleton plays important roles in cell physiology being responsible for chromosomal segregation during mitosis, providing a mechanical support to dividing cells, contributing to maintain cell shape and spatially organizing cell proteins and organelles in cell cytoplasm. Moreover, cytoskeleton is involved in cell motility, membrane trafficking, signal transduction, and cell adhesion. In addition, cytoskeletal proteins can transduce and amplify membrane receptor-captured signals, transmitting the information to the nucleus and finally regulating gene expression [2, 9, 10]. Considering all these observations, it appears easy to understand why cytoskeleton disorganization could compromise a lot of cell functions leading, in some cases, to cell death. It is well known that microgravity exposure could strongly influence cytoskeleton organization [10–17] and it is commonly accepted that cellular tensegrity alteration in microgravity exposed cells could explain, at least in part, the conversion of a mechanical cue into a biological response. In this regard, recent studies have revealed the importance of cytoskeletal integrity, such as F-actin and microtubules, in the physiological specific aspects of autophagy, and some papers described the capability of microgravity to induce autophagy in living cells [18–22]. Autophagy is an important housekeeping physiological process that is involved in cellular remodeling during development, elimination of aberrant organelles, or misfolded proteins and in the recycling of unnecessary cellular components to compensate for the limitation of nutrients during starvation. It is of interesting notice that this biological process is highly conserved from yeast to mammals. Despite several studies suggested a tumor suppressive role for autophagy, other reports support the hypothesis that this process is instead exploited by cancer cells to prime their proliferation and promote their survival [23–27].

Microgravity condition is a stressful change in the physical microenvironment for living cells; however, they seem to be able to adapt to this change of gravitational force since in the major part of studies available in the literature, the behavioral cellular modifications induced by microgravity are transient. This observation has led to the intriguing hypothesis that cells, in response to gravity changes, react triggering adaptive biological processes and autophagy could be one of them.

Testicular cells appear to be sensitive to microgravity: it has been demonstrated, in fact, that testicular function is impaired by microgravity exposure [28–34]. Moreover, some in vitro observations revealed that microgravity influences cell proliferation, apoptosis, and testosterone secretion of testicular organ cultures [35, 36]. In addition, microgravity condition has differentiating effect in cultured spermatocytes and influences germ cell survival [37, 38]. This effect on male germ cell lineage has triggered the hypothesis that also testicular cancer germ cells could be altered by microgravity condition. For this reason, we decided to study the effect of microgravity on TCam-2 cells that are the only accredited seminoma cell line [39–42]. These cells have been recently characterized at molecular and biochemical level [43–51] and thus represent a good tool to investigate male germ cell behavior modification in response to a mechanical force modification. In this paper we report, for the first time, cytoskeletal modifications and the activation of autophagic process induced by acute exposure to microgravity in TCam-2 cell line.

## 2. Materials and Methods

### 2.1. Random Positioning Machine

The random positioning machine (RPM; desktop RPM, Dutch Space, Leiden, the Netherlands), we used in the investigation, is a particular kind of 3D clinostat. It consists of two independently rotating frames. One frame is positioned inside the other giving a very complex net change of orientation to a biological sample mounted in the middle. The degree of microgravity simulation depends on angular speed and on the inclination of the disk. These tools do not actually eliminate the gravity but it is a microweight simulator based on the principle of “gravity-vector averaging”: it allows you to apply a 1 g stimulus omnidirectionally rather than unidirectionally and the sum of the gravitational force vectors tends to zero. Effects generated by the RPM are comparable to those of the real microgravity, provided that the direction changes are faster than the response time of the system to gravity field. The desktop RPM we used has been positioned within an incubator (for maintaining temperature, CO_2_, and humidity levels) and connected to the control console through standard electric cables.

### 2.2. TCam-2 Cell Cultures

The TCam-2 human cell line was derived in 1993 from a primary testicular tumor sample of pure classical seminoma [42]. TCam-2 cells were cultured in RPMI 1640 (Lonza) supplemented with 10% fetal bovine serum (FBS, Lonza) and penicillin/streptomycin (Invitrogen) at 37°C in a humidified atmosphere with 5% carbon dioxide [41]. The time 0 plating cell density is 3 × 10^4^/cm^2^. As described in the paragraph above, microgravity condition was simulated using the random positioning machine (RPM). Experiments were performed on cells cultured for 24 and 48 hours at 1 g or in RPM, after additional 24 hours of preplating on glass slides or IBIDI microscopy chambers (IBIDI, 80826). Glass slides were silicone fixed to the culture dishes at least 48 hours before plating. Cell culture dishes, in both 1 g and RPM culture conditions, were completely filled with fresh culture medium in order to avoid air bubbles and to minimize liquid flow, thus making negligible the effects of both buoyancy and shear stress during rotation.

### 2.3. Proliferation, Apoptosis, and Autophagy Quantification

Cells cultured at 1 g or under microgravity conditions (as described above) were fixed in 4% paraformaldehyde (PFA) in phosphate buffered saline (PBS) 1X for 10 minutes at 4°C and permeabilized with 1% bovine serum albumin (BSA), 0.1%-Triton X-100 in PBS 1X for 1 hour at room temperature (RT). Nonspecific antibody binding was blocked with glycine 1 M pH 8.8 and with 1% BSA, 0.1% Triton X-100, and 5% donkey serum (Jackson ImmunoResearch Laboratories) in PBS 1X. Cells were incubated overnight (ON) in PBS 1X added with 1% BSA/0.1% Triton X-100 at 4°C with the following primary antibodies: anticleaved Caspase-3 (Cell Signaling, rabbit polyclonal #9661, 1 : 200 dilution), anti-p-histone H3 (Santa Cruz Biotechnology, mouse monoclonal sc-374669, 1 : 50 dilution), or anti-LC3 (Sigma-Aldrich, L7543 1 : 120 dilution). After rinsing, samples were incubated with the opportune secondary antibody (FITC-conjugated donkey anti-rabbit 711-095-152 or donkey anti-mouse 715-095-150 IgG, Jackson ImmunoResearch Laboratories, 1 : 200 dilution) in PBS 1X for 90 min at RT. In negative controls primary antibody was omitted. After secondary antibody incubation, samples were washed and mounted in buffered glycerol (0.1 M, pH 9.5). All experiments were performed at least in triplicate.

For proliferation and apoptosis analyses, samples were photographed with a Zeiss fluorescence microscope (Axioscope) and positive cells were counted. For LC3 immunolocalization a Leica confocal microscope (Laser Scanning TCS SP2) equipped with Ar/ArKr and He/Ne lasers was used. Images were acquired utilizing the Leica confocal software. The laser line was at 488 nm for FITC excitation. The images were scanned under a 20x objective or 40x oil immersion objective. In order to get a quantitative analysis of fluorescence, optical spatial series, each composed of 23/25 optical sections with a step size of 2 *μ*m, were performed in areas in which cells reached confluence both in nonrotated and in RPM cultured samples. The fluorescence intensity was determined by the Leica confocal software, using the following parameters: the maximum amplitude of fluorescence (MAX Amplitude), the sum of intensity (SUM (I)), and the mean amplitude of fluorescence intensity (MEAN (A)), of LC3 positive areas. The MAX Amplitude represents the maximum intensity of fluorescence of each series. The SUM (I) represents the total amount of fluorescence intensity recovered within the entire *z*-axis of each series. The MEAN (A) represents the arithmetical mean of fluorescence intensity recovered within the entire *z*-axis of each series. We analyzed 12 equivalent sized regions (regions of interest (ROI)) for each experiment both in 1 g and in RPM culture conditions (36 total ROI for each experimental condition).

### 2.4. Western Blotting of LC3 Autophagy Marker

Cells cultured at 1 g and in RPM condition for 24 and 48 hours were lysed in RIPA buffer (Sigma-Aldrich). Samples were then clarified by centrifugation at 10000 rpm for 10 min. Equivalent amount of protein (10 *μ*g) from each sample was electrophoretically resolved on 12.5% precast SDS-polyacrylamide gels (ExcelGel, GE Healthcare Biosciences) using horizontal apparatus (Pharmacia Biotech, Uppsala, Sweden). Then, separated proteins were electrotransferred onto nitrocellulose membranes (Schleicher & Schuell) by a semidry system (Novablot, Pharmacia Biotech). Membranes were blocked with 3% nonfat milk in PBS and then were incubated (ON at 4°C) with the LC3B monoclonal antibody (1 : 2000; Sigma). After extensive washing with PBS containing 0.1% tween-20 (TBST), blots were incubated with 1 : 2000 dilution of HRP-conjugated secondary antibody (Amersham Biosciences) for 1 hour at RT. Immunopositive bands were detected with a chemiluminescence's detection system (GE Healthcare Biosciences). To check for equal loading of the gel, membranes were stripped and reprobed with mouse anti-*β*-actin antibody (1 : 20000, Sigma) and with anti-GAPDH antibody (1 : 1000, Cell Signalling Technology). Densitometric analysis was performed with the Quantity One software (BioRad Laboratories).

### 2.5. F-Actin and Tubulin Distribution Pattern

For F-actin visualization Rhodamine Phalloidin (Invitrogen Molecular Probes Eugene, 1 : 40 dilution) was used. Cells were fixed in 4% paraformaldehyde (PFA) in PBS for 10 minutes at 4°C and then permeabilized with cold ethanol : Acetone 1 : 1 for 10 minutes at 4°C. After rinsing, cells were incubated with Rhodamine Phalloidin for 25 min in the dark. Cells were then washed in PBS and mounted in buffered glycerol (0.1 M, pH 9.5).

Cell height analysis (*z*-axis) was performed using the confocal microscope already described (Leica IRE SP2, Laser Scanning TCS SP2) equipped with Ar/ArKr and He/Ne lasers. Images of the optical sections were acquired using the Leica confocal software. The Laser Line was at 543 nm for TRITC excitation. Images were scanned under a 40x oil objective. In order to evaluate cell height three different experiments were performed using cells cultured 1 g and in RPM conditions. For each experiment 4/5 optical spatial series with a step size of 2 *μ*m were recovered and a total of at least 80 optical sections were analyzed for each experimental condition. Cell height of the examined samples was calculated using Leica confocal software.

For microtubules localization immunofluorescence experiments, using anti-*α*-tubulin (Biomeda, mouse monoclonal V10178, 1 : 75 dilution) as primary antibody, were performed. The protocol used is the same already described in the paragraph above. Donkey anti-mouse (715-095-150 IgG, Jackson ImmunoResearch Laboratories, 1 : 200 dilution), as secondary antibody, was used. Samples were then observed using both fluorescence microscope (Axioscope, Zeiss) and confocal microscope (Leica).

### 2.6. Scanning Electron Microscopy

Samples were fixed in Glutaraldehyde 2.5% in cacodylate buffer 0.1 M pH 7.3 ON and then postfixed with 1% osmium tetroxide in cacodylate buffer 1 M, dehydrated with increasing ethanol percentage (30–90% in water for 5 min, twice 100% for 15 min), treated in Critical Point Dryer (EMITECH K850), sputter coated with platinum-palladium (Denton Vacuum DESKV), and observed with Supra 40 FESEM (Zeiss).

### 2.7. Statistical Analysis

All experiments were performed at least in triplicate. All quantitative data are presented as the mean value ± standard error (SEM). Student's *t*-test and ANOVA test for multigroup comparison were carried out, when appropriate, to evaluate the significance of differences. The significance level was fixed at a *P* value <0.05.

## 3. Results and Discussion

### 3.1. Microgravity Does Not Affect TCam-2 Cell Proliferation and Apoptosis

**Figure 1:**
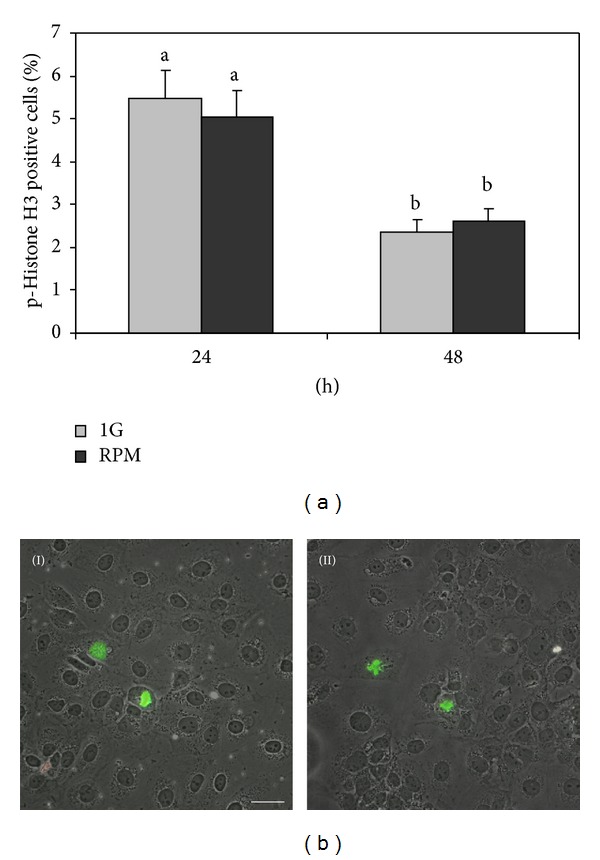
RPM exposure does not influence TCam-2 cell proliferation. (a) Graphical representation of the percentage of proliferating cells (p-histone H3 positive cells) at 24 and 48 hours of culture. No differences were observed between TCam-2 cells cultured at 1 g or in RPM culture conditions. Data are expressed as the mean ± SEM. Same letters indicate no statistical difference. Different letters indicate *P* < 0.05. (b) Representative images of TCam-2 cells cultured for 24 hours at 1 g (I) and in RPM condition (II) after p-histone H3 immunofluorescence. Bar, 50 *μ*m.

Microgravity exposure is known to influence cell proliferation and apoptosis in normal and cancer cells [52]. In order to asses proliferation rate of TCam-2 seminoma cells, maintained at 1 g or in RPM culture conditions for 24 and 48 hours, we performed immunofluorescence analyses of the M-phase marker p-histone H3. We observed that, actually, this acute microgravity exposure does not affect the number of mitotic cells at all the culture times considered (Figure 1). Literature data have demonstrated that TCam-2 cells do not have a high proliferation rate (58 hours doubling time) when compared with JKT1 (27 hours doubling time), that is, another germ cell tumor cell line [40]. Since the percentage of proliferating cells we expect in the time frame of 24 and 48 hours is not high, we can hypothesize that this altered gravitational stimulus is not long enough to determine a modification of cell proliferation in this particular cell line. Interestingly after 48 hours of culture the number of mitotic cells decreases significantly, in a similar amount, both in 1 g and in RPM cultured samples (Figure 1), indicating that cell proliferation, in this particular cell line, starts to be inhibited by cell-to-cell contact even if these cells are cancer cells. It has to be noticed that we chose to plate cells at high density in order to let them attach each other before the RPM exposure and react, thanks to their tensional forces, to the changes of gravitational field. Due to the high density of plating, at the end of the longer culture time we analyzed, cell culture dishes are crowded of cells so it appears not possible to prolong more the culture without detaching and replate cells. To this regard it is fair to say that we cannot exclude that TCam-2 cell proliferation might be altered by RPM exposure if they would have been cultured at a different density.

**Figure 2:**
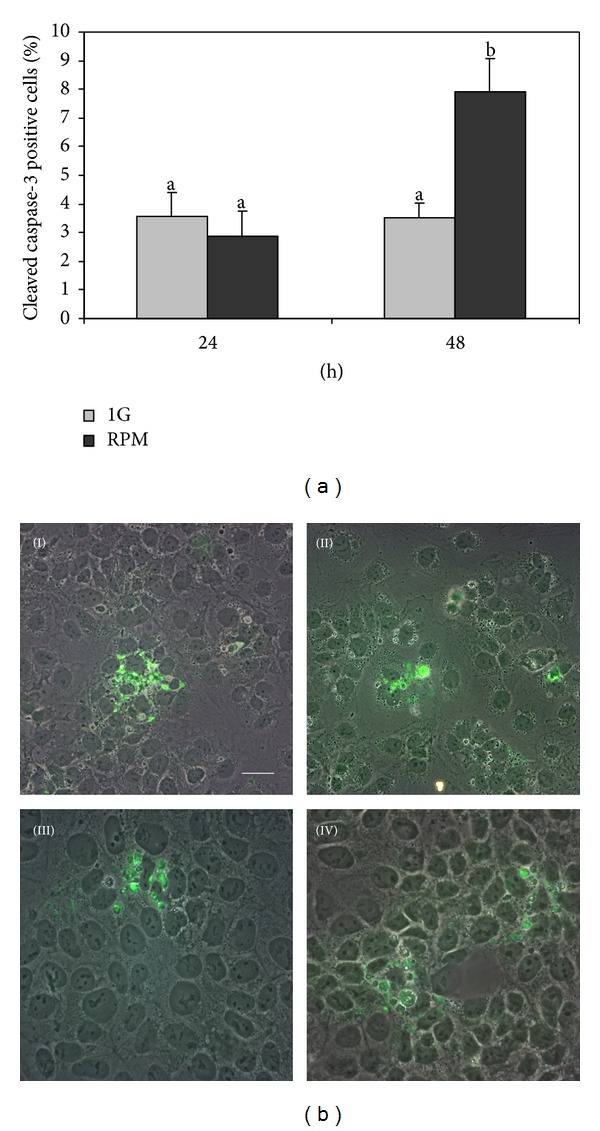
RPM exposure and TCam-2 cell apoptosis. (a) Graphical representation of the percentage of apoptotic cell number (anticleaved Caspase-3 positive cells). No differences were observed between TCam-2 cells cultured for 24 hours at 1 g or in RPM culture conditions. On the contrary a slight increase in apoptotic cell percentage is observed after 48 hours of culture. Data are expressed as the mean ± SEM. Same letters indicate no statistical difference. Different letters indicate *P* < 0.01. (b) Representative images of 1 g (I, III) and RPM (II, IV) exposed TCam-2 cells in 24 (I, II) and 48 (III, IV) hours of culture after cleaved Caspase-3 immunofluorescence. Bar: 50 *μ*m (I and II); 35 *μ*m (III and IV).

To test whether microgravity would be able to modify TCam-2 cell apoptosis, we performed immunofluorescences for the active fragment of the apoptosis marker Caspase-3. We found that the change of gravity vector does not affect the number of apoptotic cells after 24 hours of culture (Figure 2). However, it has to be noticed that, after 48 hours of culture, the number of apoptotic cells increases significantly in the RPM cultured samples, even if the large majority of cells appear to tolerate this mechanical stress (Figure 2) and to survive. The latter observation indicates that a small part of TCam-2 cells appears more sensible to the change of gravity vector, when the mechanical stimulus is prolonged a bit, but this sensibility does not seem related to mechanical cell stability because, due to the high density of plating, all cells are stably attached to each other and to the substrate. In addition, apoptotic cells are observable uniformly dispersed in the culture dish. On the basis of this observation, we hypothesized that TCam-2 cells need to trigger rescue processes that let them survive after a prolonged change of gravity vector. Possibly, rescue processes are not correctly induced or exploited by the whole population of TCam-2 cells and this hypothesis may explain why a small percentage of them appears not able to survive to the change of gravity. The change of physical forces is sensed by the cells through their cytoskeleton components and one of the first features that reveal a cytoskeletal modification is the change in the plasma membrane morphology. We studied first membrane surface and cytoskeletal modifications, due to RPM exposure, to be sure the TCam-2 cells are able to sense and modify their shape in response to this mechanical stress. Then we evaluated, in the same culture conditions, the autophagic process modulation in response to RPM exposure, since autophagy is the most known biological rescue mechanism that let cell to change rapidly and survive to sudden microenvironmental changes.

### 3.2. Microgravity Strongly Influences TCam-2 Cell Membrane Surface

**Figure 3:**
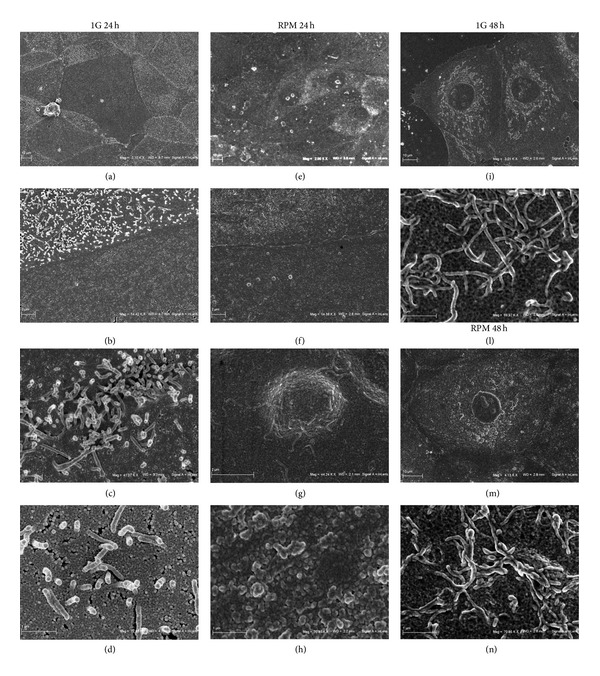
Microgravity effect on TCam-2 cell membrane surface. Scanning electron microscopy pictures with increasing magnification showing cell membrane surface morphology of TCam-2 cells cultured for 24 (a, b, c, and d) and 48 hours (i, l) at 1 g or for 24 (e, f, g, and h) and 48 hours (m, n) in RPM culture conditions. In (a) white asterisks indicate TCam-2 cells with smooth membrane surface while the other TCam-2 cells of the same image are characterized by the presence of microvilli-like structures. In (b) the boundary between one smooth membrane and one microvilli membrane presenting cells is reported. (c) and (d) represent higher magnifications of the microvilli-like structures of TCam-2 cells maintained at 1 g. In (e), (f), (g), and (h) it is well evident that, in RPM cultured cells, membrane surface is more similar in all the cells and it is difficult to clearly identify the two cell populations. In particular in (h) it is possible to observe that microvilli-like structures appeared collapsed in RPM exposed TCam-2 cells. The morphological appearance of cell surface (i, m) and microvilli-like structures (l, n) appeared indistinguishable in 1 g (i, l) and RPM exposed cells (m, n) after 48 hours of culture.

To study if the alteration of the mechanical forces acting on TCam-2 cells during microgravity simulation may modify cell membrane surface morphology, samples were analyzed by scanning electron microscopy. We observed the presence of two morphologically distinguishable cell populations in the 1 g cultured samples: one has smooth membrane surface and the other one is characterized by the presence of membrane expansions morphologically similar to microvilli (Figure 3). Noteworthy, we found that microgravity strongly affects membrane surface appearance after 24 hours of culture: microvilli appeared collapsed and the differences between the two cell populations are less evident (Figure 3). It is of interesting notice that cell microvilli are considered to be an important site of mechanotransduction both in sensory specialized cells and not-sensory cells [53]. After 48 hours of culture the membrane surface differences appear recovered and microvilli-like structures appear reconstituted in RPM cultured samples (Figure 3). On the basis of these observations, we hypothesized that cell mechanosensor-system was transiently altered by RPM exposure and this strongly suggested the occurrence of cytoskeleton remodeling due to an acute exposure to gravitational vector change.

### 3.3. Microgravity Induces TCam-2 Cytoskeleton Remodeling

**Figure 4:**
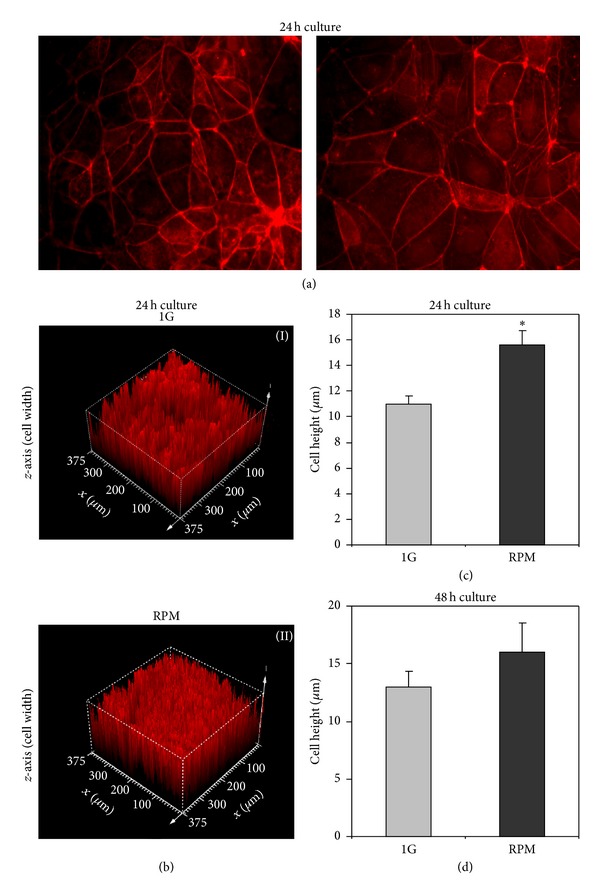
Simulated microgravity influences TCam-2 cell height. (a) Rhodamine-phalloidin staining of TCam-2 cells showing F-actin distribution pattern after 24 hours of culture at 1 g (I) or under RPM (II) conditions. Bar, 20 *μ*m. (b) Representative images of cell height obtained using the Leica confocal software, of samples cultured for 24 hours at 1 g (I) or in RPM (II) conditions. (c) Graphical representation of cell height obtained by confocal microscopy analysis on 1 g and RPM exposed cells after 24 hours of culture (*15.62 ± 1, 10 *μ*m versus 11.0 ± 0.66 *μ*m; *P* < 0.001). Data are expressed as the mean ± SEM. (d) Graphical representation of cell height obtained by confocal microscopy analysis on 1 g and RPM exposed cells after 48 hours of culture (13.02 ± 1.32 *μ*m versus 16.02 ± 2.49 *μ*m, resp.). Data are expressed as the mean ± SEM. The values are not statistically significant.

A huge amount of literature data demonstrated that microgravity is able to influence cell cytoskeletal architecture, promoting cell morphofunctional alterations [54]. In the light of these observations and on the basis of our scanning electron microscopy data, we decided to evaluate the possible effects of simulated microgravity on TCam-2 microfilament and microtubule organization. Herein, we report microfilament distribution pattern analyzed by F-actin staining of TCam-2 cells cultured at 1 g or in RPM culture conditions. Even if no apparent significant alterations in the actin cytoskeleton organization were found both in 24 (Figure 4(a)) and 48 hours of culture (not shown), a more detailed analysis by confocal microscopy using Leica confocal software allowed us to evaluate cell height (cell *z*-axis) (Figures 4(b), 4(c), and 4(d)) in all the considered experimental conditions. We observed that simulated microgravity significantly increases TCam-2 cell height after 24 hours of RPM exposure with respect to 1 g cultured cells (15.62 ± 1.10 *μ*m versus 11.0 ± 0.66 *μ*m; *P* < 0.001) indicating that RPM culture condition was able to modify TCam-2 cell shape. Noteworthy, after 48 hours of culture the differences in cell height in 1 g and RPM cultured cells are no more statistically significant (Figure 4(d)), indicating that TCam-2 cells are able to recover rapidly after the exposure to this mechanical stress. The latter observation appears consistent with the reported recovery of surface membrane microvilli-like structures after 48 hours of RPM exposure (Figure 3).

**Figure 5:**
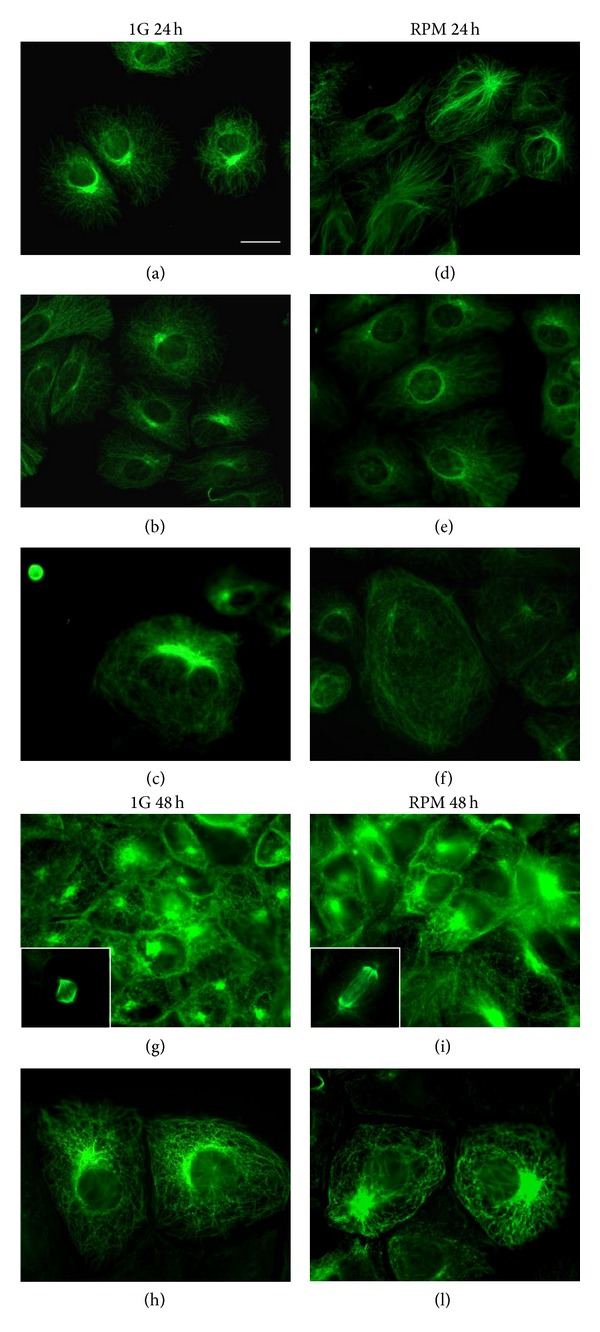
Microtubule distribution pattern in TCam-2 cells exposed to simulated microgravity. Immunodetection of *α*-tubulin in TCam-2 cells cultured for 24 hours (a, b, c, d, e, and f) and 48 hours (g, h, i, and l) at 1 g (a, b, c, g, and h) or under RPM conditions (d, e, f, i, and l). In images (g) and (i), in the white box, representative images of mitotic spindles are also shown. Bar, 20 *μ*m.

Microtubule distribution pattern was studied by anti-*α*-tubulin immunofluorescence staining. After 24 hours of culture, we observed that microtubule distribution is altered in TCam-2 cells exposed to RPM culture condition: centriolar polarization is much less visible in these samples and microtubules appear to be distributed in an apparently random manner within the cells (Figure 5). Microtubules are key regulators of membrane trafficking; organelle distribution inside the cells and together with actin microfilaments seems to regulate autophagosome formation [55–57]. In addition it is of interesting notice that LC3, the marker protein of the autophagic process, is a microtubule associated protein (MAP). As well as actin filaments, after 48 hours of culture the microtubule distribution pattern appears recovered in RPM exposed samples since it is not possible to observe significant differences between 1 g and RPM cultured cells. These observations again clearly indicate the capability of TCam-2 cell to sense the change of physical forces in their microenvironment and also to recover rapidly from this physical stress. These data strongly suggest the trigger of rescue mechanisms due to TCam-2 RPM exposure.

It is worth mentioning that the reported microtubule alteration does not appear to significantly alter the proper formation of the mitotic spindle (Figure 5(g) white box). This observation is consistent with the results reported in Figure 1 in which we observed that TCam-2 cell proliferation does not appear to be affected by RPM exposure.

### 3.4. Microgravity Induces TCam-2 Cell Autophagy

Some papers in the literature reported that, in other cellular systems, microgravity is involved in autophagy induction [18–20] and, as previously stated, cytoskeleton plays important roles in autophagy regulation [22]. In particular, in mammals, microtubules appear to be involved in the fusion of autophagosome with late endosome and to bind and transport autophagosomes, once terminally completed. The role of actin filaments on mammalian autophagy process regulation is still a matter of debate, but it is worth mentioning that microfilaments depolymerization agents are able to block autophagosome formation.

**Figure 6:**
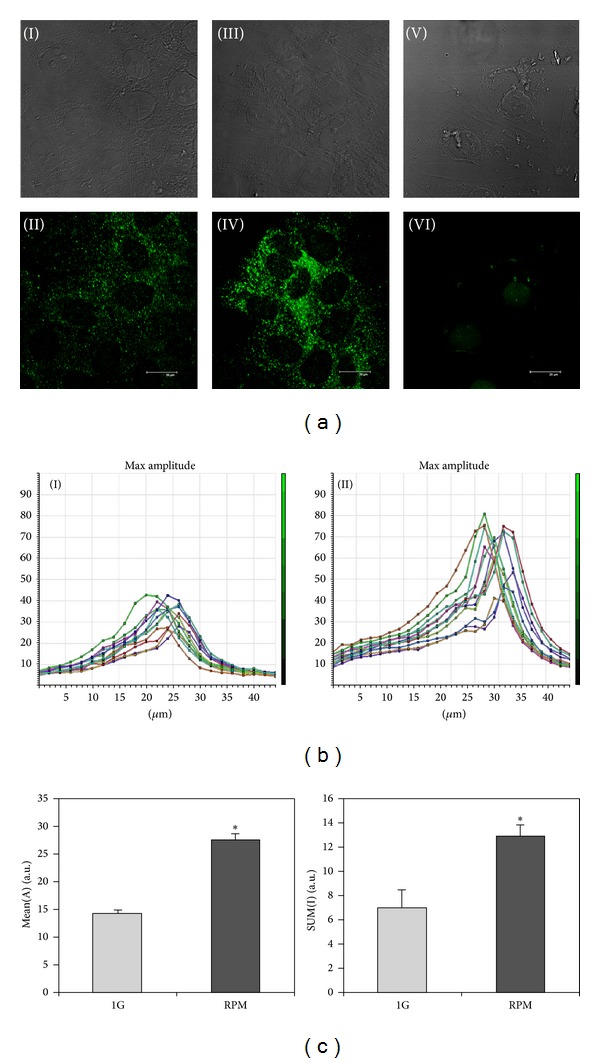
Autophagy induction in TCam-2 cells exposed to microgravity. (a) Immunodetection of LC3 in TCam-2 cells cultured for 24 hours at 1 g (II) or under RPM (IV) conditions. In VI LC3 immunodetection of TCam-2 cells cultured in RPM condition for 48 hours is reported. In I, III, and V the respective bright fields are shown. (b) Stack profile of 24 hours of culture representative experiment showing the maximum amplitude (MAX Amplitude) of fluorescence in 12 regions of interest (ROI), randomly drawn in an area in which the cells reached confluence, in nonrotated (I) and RPM cultured samples (II). It is evident an increase of maximum amplitude of fluorescence in microgravity exposed samples (II) with respect to the 1 g-cultured cells (I). (c) MEAN (A) (*27.62 ± 1.04 versus 14.34 ± 0.59; *P* < 0.001) and SUM (I) (*12.92 ± 0.85 versus 6.95 ± 1.52; *P* < 0.05) confirm an increase of LC3 positivity in RPM exposed sample with respect to 1 g cells after 24 hours of culture. Data are expressed as the mean ± SEM.

**Figure 7:**
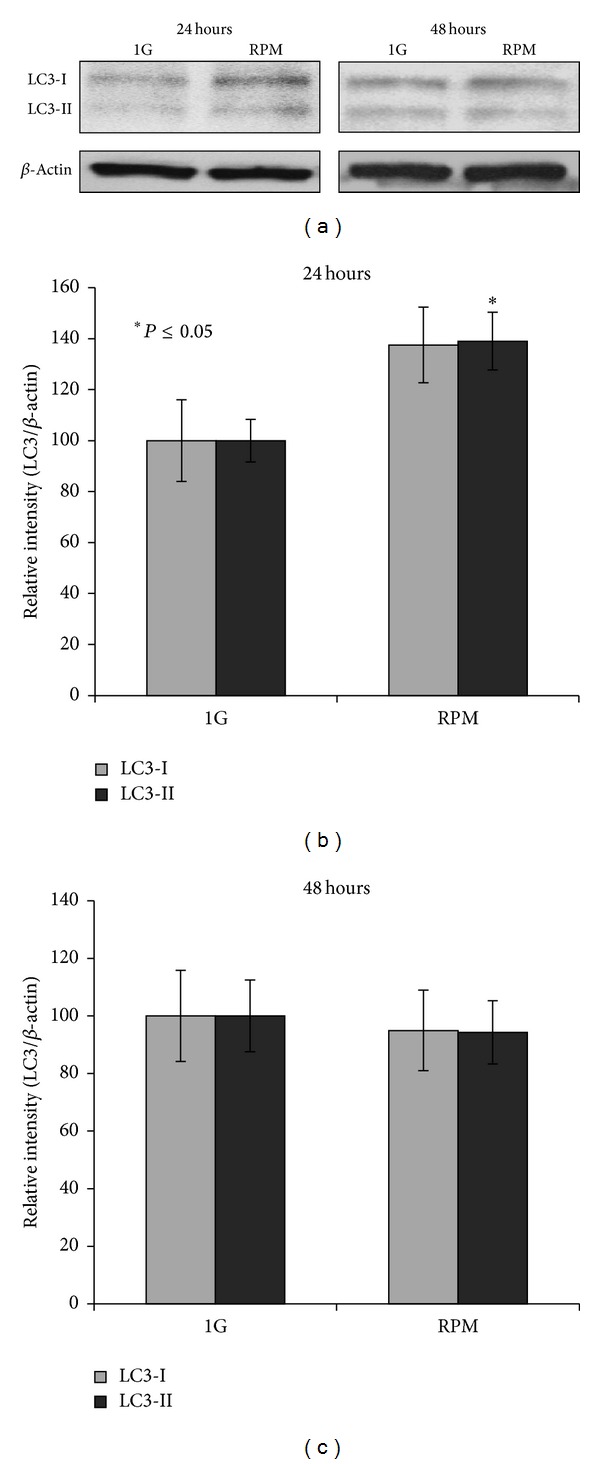
Western blot analysis of LC3 autophagy marker. (a) Representative images of the bands revealed by anti-LC3 western blot analysis on 24 and 48 hours cultured samples. As expected anti-LC3 antibody detected both the LC3 isoforms (LC3-I cytosolic isoform; LC3-II autophagosomal membrane-conjugated isoform). (b) Graphical representation summarizing the densitometric analysis of the LC3-I and LC3-II bands, normalized versus *β*-actin in 24 hours cultured samples. Data are expressed as the mean ± DS. *versus 1 g *P* < 0.05. (c) Graphical representation summarizing the densitometric analysis of the LC3-I and LC3-II bands, normalized versus *β*-actin in 48 hours cultured samples. Data are expressed as the mean ± DS. The values are not statistically significant.

TCam-2 cells cultured at 1 g and in RPM conditions were immunostained to detect the autophagic marker LC3. As shown in Figures 6(a)(II) and 6(a)(IV), LC3 is detectable both in 1 g and in RPM cultured samples and it is mainly localized in cytoplasmic vesicles. Interestingly, the number of these LC3 positive vesicles appears strongly increased in TCam-2 cells exposed to microgravity conditions (Figure 6(a)(IV)) with respect to 1 g cultured cells (Figure 6(a)(II)) after 24 hours of culture. Moreover, a quantitative analysis, carried out using the Leica confocal software, allows us to quantify the fluorescence intensity increase of LC3 stained cells exposed to simulated microgravity (Figures 6(b) and 6(c)). In particular, Figure 6(b) shows a stack profile of 12 regions of interest (ROI) of a representative experiment both in 1 g (I) and in RPM cultured samples (II). The two groups of peaks reported in this figure represent the Max amplitude of fluorescence detected by the confocal microscope from the beginning to the end of the sample (total *z*-axis). It is well evident that Max amplitude of fluorescence is increased in simulated RPM exposed samples. We evaluated also the SUM (I) and the MEAN (A) of fluorescence. Consistent with the data reported in Figure 6(b), we observed also an increase of both the SUM (I) and the MEAN (A) in RPM cultured cells after 24 hours of culture (Figure 6(c)). According to the described confocal quantitative analyses, western blots performed with the anti-LC3 antibody showed that, besides the increase of LC3-I protein amount, LC3-II (the LC3 active isoform) protein content is increased in RPM with respect to 1 g cultured samples (Figure 7). Same results were obtained normalizing the LC3 bands versus *β*-actin (Figure 7) and versus GAPDH signal (not shown). Autophagy induction is a naturally transient process: this phenomenon is called autophagic flux [58], since, when it works, autophagy protein machinery has to be degraded via lysosomes or proteasome together with the portion of the cell that needs to be eliminated. On the contrary, when autophagy is blocked, the autophagy protein machinery is not degraded and is maintained at high level in the cytoplasm. In our samples, after 48 hours of culture autophagy active protein LC3-II, together with LC3-I, appears quantitatively similar in 1 g and RPM cultured cells, demonstrating that autophagy is restored at the same level with respect to 1 g culture condition. Same results were obtained normalizing the LC3 bands versus *β*-actin (Figure 7) and versus GAPDH signal (not shown). Consistent with this observation, the LC3 cytoplasmic fluorescence is lowered in the RPM exposed cells demonstrating that autophagy was not blocked by this mechanical stress (Figure 6(a)(VI)). It has to be mentioned that LC3-II protein is present at basal level at 24 and 48 hours of culture as well as cytoplasmic LC3 dots, even in cells cultured at 1 g, indicating that autophagy is a housekeeping process that works in TCam-2 cells even in control samples and suggesting that this cancer cell line may exploit autophagy as a survival mechanism.

There is a common agreement indicating that there is a relationship between autophagy and apoptosis: when autophagy is not able to rescue cell from microenvironmental changes, apoptotic process is triggered. On the light of this theory we might interpret the small increase in the apoptotic index at 48 hours of culture in RPM cultured samples (Figure 2) as the autophagy efficiency threshold or the limit of autophagy efficiency in the rescue of cell survival after mechanical stress exposure.

All together these qualitative and quantitative analyses allow us to conclude that microgravity is able to positively modulate the autophagic process in TCam-2 seminoma cell line. Autophagy induced in TCam-2 cells by Estrogen exposure through ER*β* activation was recently reported [59]. Herein we reported, for the first time, autophagy induced in TCam-2 cells by a mechanical cue (or, more precisely, by a removal of a mechanical stimulus) instead of a biochemical one. The analysis of the autophagy related pathways induced by RPM exposure and the direct role of microtubules and microfilaments in this process, as well as the other possible biological meanings of RPM induced TCam-2 autophagy, deserves further investigations.

## 4. Conclusions

Gravitational biology could be considered part of mechanobiology, the science that investigates the impact of forces on living organisms. At cellular level, cytoskeleton elements are likely candidates for force sensing and transduction processes. These biomechanical properties of cell cytoskeleton explain the capability to propagate a mechanical stimulus over long distances in living tissues and represent the basis of the intriguing hypothesis that many, if not all, reported changes in ion fluxes, protein phosphorylation, membrane potential changes, and so forth are indeed provoked by a mechanical modification somewhere within the cell or on its membrane [60, 61]. This paper is in line with this theory and adds experimental data supporting the importance of mechanotransduction and cell behavior. In this paper, in fact, we reported the effects of the exposure to changes of gravity vector on TCam-2 seminoma cells. In this experimental model, simulated microgravity is able to induce TCam-2 cell surface modifications and microvilli-like structure alteration. Moreover, microtubules and microfilaments organization result to be influenced by microgravity: (a) TCam-2 cells show actin cytoskeleton remodeling and cell height increase; (b) centriolar polarization becomes much less visible in these samples and microtubules appear to be distributed in an apparent random manner within the cells. All these modifications appear to be transient, indicating that cells modify their cytoskeletal components in response to gravitational force change, but that are also able to recover their shape when the gravitational change is prolonged. Interestingly, RPM exposure is able to induce TCam-2 cell autophagy. The latter observation allows us to hypothesize that TCam-2 cells are able to rapidly respond to acute exposure to microgravity, inducing adaptive biological processes such as autophagy, that probably allow them to survive in the changing physical microenvironment. Since autophagy is considered a biological survival mechanism the apoptosis induction in a small percentage of TCam-2 cells after 48 hours of culture might be speculated as the limit in the efficiency of this survival process. All together these data provide evidences of TCam-2 sensitivity to changes of gravitational force direction and lay the groundwork to further studies on TCam-2 cell autophagy and its biological meaning.
